# The Potential Role of R4 Regulators of G Protein Signaling (RGS) Proteins in Type 2 Diabetes Mellitus

**DOI:** 10.3390/cells11233897

**Published:** 2022-12-02

**Authors:** Xiaohong Zhang, Hongyan Lv, Juan Mei, Bingyuan Ji, Shuhong Huang, Xuezhi Li

**Affiliations:** 1 Shandong Collaborative Innovation Center for Diagnosis, Treatment and Behavioral Interventions of Mental Disorders, Institute of Mental Health, Jining Medical University, Jianshe South Road No.45, Rencheng District, Jining 272013, China; 2 Shandong Key Laboratory of Behavioral Medicine, School of Mental Health, Jining Medical University, Jianshe South Road No.45, Rencheng District, Jining 272013, China; 3 Department of Pediatrics, Jining No.1 People’s Hospital, Jiankang Road No.6, Rencheng District, Jining 272011, China; 4 Institute of Precision Medicine, Jining Medical University, Hehua Road No.133, Taibai Lake District, Jining 272067, China; 5 Central Laboratory, Shandong Provincial Hospital Affiliated to Shandong First Medical University, Jingwuweiqi Road No.324, Huaiyin District, Jinan 250021, China; 6 Department of Biochemistry and Molecular Biology, School of Clinic and Basic Medicine, Shandong First Medical University and Shandong Academy of Medical Sciences, Qingdao Road No.6699, Huaiyin District, Jinan 250117, China

**Keywords:** type 2 diabetes mellitus, insulin, Gα, regulators of G protein signaling

## Abstract

Type 2 diabetes mellitus (T2DM) is a complex and heterogeneous disease that primarily results from impaired insulin secretion or insulin resistance (IR). G protein-coupled receptors (GPCRs) are proposed as therapeutic targets for T2DM. GPCRs transduce signals via the Gα protein, playing an integral role in insulin secretion and IR. The regulators of G protein signaling (RGS) family proteins can bind to Gα proteins and function as GTPase-activating proteins (GAP) to accelerate GTP hydrolysis, thereby terminating Gα protein signaling. Thus, RGS proteins determine the size and duration of cellular responses to GPCR stimulation. RGSs are becoming popular targeting sites for modulating the signaling of GPCRs and related diseases. The R4 subfamily is the largest RGS family. This review will summarize the research progress on the mechanisms of R4 RGS subfamily proteins in insulin secretion and insulin resistance and analyze their potential value in the treatment of T2DM.

## 1. Introduction

The pathogenesis of T2DM is primarily characterized by impaired insulin secretion (IIS) and peripheral insulin resistance (IR) (or decreased insulin sensitivity). Insulin signaling regulates glucose and lipid metabolism mostly via action on the liver, adipose tissue, and skeletal muscle. IR stands for impaired insulin sensitivity caused by the disruption of various molecular pathways [1,2]. As human cells predominantly rely on glucose as a source of energy, and the cellular uptake of glucose is controlled by insulin, IIS or IR can be risk factors for numerous metabolic diseases in the liver, cardiovascular system, nervous system, etc. [1,2]. However, IIS and IR are best known to be associated with T2DM. The underlying mechanisms of IIS and IR are not yet clearly illustrated. A number of credible theories have been proposed, such as insulin receptor mutations, endoplasmic reticulum stress, oxidative stress, and inflammation.

GPCRs are proposed as important therapeutic targets for T2DM, and increasing evidence suggests that many GPCRs are involved in insulin secretion or uptake [3,4]. In addition to designing agonists/antagonists that directly induce/inhibit GPCR activation, RGSs are also popular targeting sites for modulating the signaling and function of GPCRs, whereas RGS proteins determine the extent and duration of cellular responses to GPCR stimulation [5]. In total, four RGS protein subfamilies—RZ, R4, R7, and R12—have been characterized since the 1990s. The R4 subfamily is the largest RGS protein subfamily, and most members have been indicated to be involved in T2DM. This review will summarize the research progress on the association and related underlying mechanisms of R4 RGS subfamily members in insulin secretion and insulin resistance and analyze their potential value in the treatment of T2DM.

## 2. Gα Protein Signaling and the RGS Superfamily

**Figure 1:**
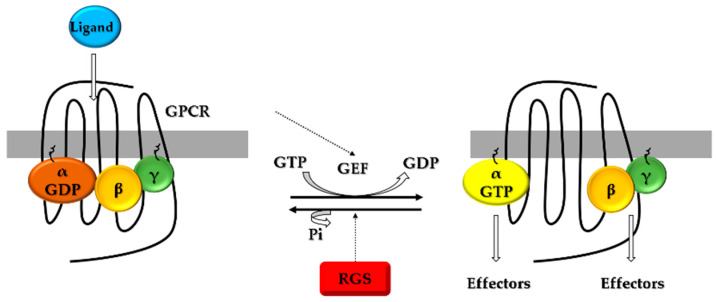
A standard model of the guanine nucleotide loop of receptor-mediated heterotrimeric G protein activation.

Heterotrimeric guanine nucleotide-binding proteins (G proteins, Gαβγ) mediate classical cell signal transduction [6]. They transduce a range of extracellular signals received from cell-surface GPCRs, such as those initiated by circulating hormones and neurotransmitters in synapses. In traditional heterotrimeric G protein activation models, extracellular ligands bind to cell-surface receptors, inducing conformational changes in the receptors [7]. The activated receptor functions as a guanine nucleotide-exchange factor (GEF), catalyzing the exchange of guanine nucleotides on the Gα subunit (GDP bound) of the Gβγ complex (Figure 1). Gα will convert from the inactive GDP-bound form to the active GTP-bound form and dissociate from the Gβγ dimer. Subsequently, the Gα·GTP and Gβγ subunits interact with different downstream effectors, respectively, and continue to propagate the signal forward. There are four families of Gα subunits in mammals (Gα_s_, Gα_i/o_, Gα_q/11_, and Gα_12/13_) that differ in terms of coupled receptors, specific downstream effectors, and net cellular responses. The Gα subunit then intrinsically hydrolyzes GTP to GDP through intrinsic GTP hydrolysis (GTPase) activity and resets the cycle [8]. Gα·GDP has a lower affinity for effectors but a higher affinity for Gβγ. As a result, G protein signaling is inactivated and the inactive GDP-binding heterotrimer (Gαβγ) is reconstituted and binds to cell-surface receptors again.

In the late 1980s, a difference was discovered between the biochemical GTPase activity of the Gα subunit and the rate of closure of the cell’s response to endogenous GPCR ligands [8]. The purified Gα subunit hydrolyzed GTP in vitro too slowly to explain the rapid recovery of the G protein-mediated biological responses. Therefore, it was hypothesized that there are other cofactors that can aid the hydrolase activity of the Gα subunit. In 1996, an RGS was discovered that binds to the Gα subunit through a characteristic “RGS box” domain consisting of about 120 amino acids [9,10,11,12,13,14]. It greatly accelerates the intrinsic GTPase activity of the Gα subunit [15,16,17] and promotes the rebinding of Gα·GDP and Gβγ subunits to receptors on the cell membrane, thereby terminating signaling to the downstream effectors (Figure 1) [18]. Thus, RGS proteins function as GAPs via directly interacting with Gα·GTP to accelerate the hydrolysis of GTP bound to Gα subunits [19,20]. In this way, RGS proteins determine the size and duration of cellular responses to GPCR stimulation.

**Figure 2:**
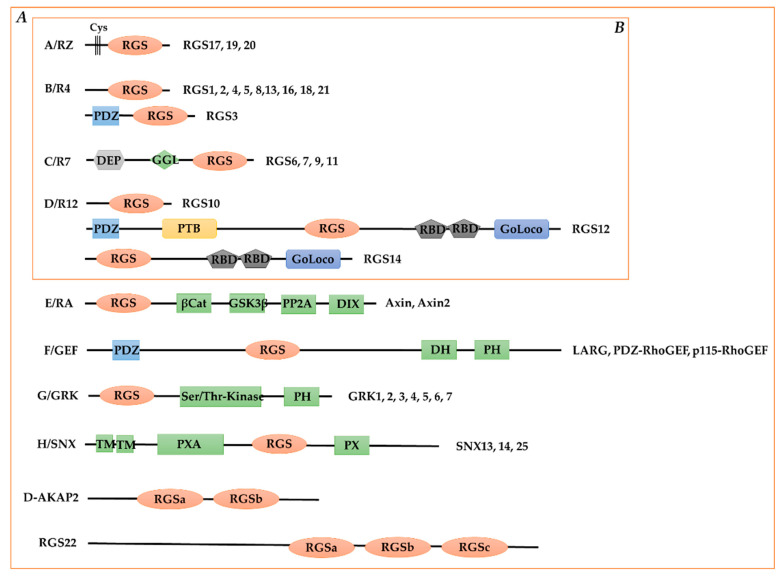
Members and their multidomain structures of the mammalian RGS protein family. (**A**) Members grouped according to David P. Siderovski and Francis S. Willard [19]. (**B**) Nomenclature according to the latest International Union of Pharmacology (IUPHAR). Members of the A/RZ subfamily, characterized by a reversibly palmitoylated N-terminal polycysteine region (“Cys”). Members of the B/R4 subfamily contain only short peptide sequences flanking the RGS box, with one notable exception (RGS3). Members of the C/R7 subfamily can bind to Gβ_5_ through the “GGL” domain. Of the three members of the D/R12 subfamily, RGS10 is the smallest, containing slightly more than one RGS box, whereas both RGS12 and RGS14 possess a tandem Ras-binding domain (RBD) and a C-terminal Gα_i/o_-Loco interaction (GoLoco) motif. RGS12 also has N-terminal PDZ (PSD95/Dlg/ZO-1 homology) and PTB (phosphotyrosine binding) domains. Axin and Axin2 (also known as Axil) are negative regulators of the Wnt signaling pathway and are contained in the E/RA subfamily. These two proteins interact with the tumor suppressor protein adenomatous polyposis coli (APC) using the RGS box. Axin and Axil also contain domains that interact with β-catenin, the kinase GSK3β, the phosphatase PP2A, and the protein Dishevelled (“DIX” domain). The F/GEF subfamily includes three RhoA-specific guanine nucleotide-exchange factors (“GEFs”) with canonical Dbl homology (DH) and pleckstrin homology (PH) domains and an N-terminal PDZ domain. The G/GRK subfamily is a family of G protein-coupled receptor kinases, and family members are recognized with an N-terminal RGS box. Members of the H/SNX subfamily have an RGS box between the phosphatidylinositol-binding (PX) and PX-associated (PXA) domains. Multiple RGS-box family members, D-AKAP2 and RGS22, reside outside of the eight established subfamilies.

The founding members of the RGS superfamily were identified in 1996 in a wide range of species: “supersensitivity to pheromone-2” (Sst2) in the budding yeast *Saccharomyces cerevisiae* [10], FlbA in the aspergillus *Emericella nidulans* [14], EGL-10 in the nematode worm *Caenorhabditis elegans* [12], and RGS1 and RGS2 from human B- and T-lymphocytes, respectively [11,13]. Subsequently, researchers have successively identified a large number of new RGS box-containing proteins in a wide range of species. They are defined by the presence of characteristic structural domains (RGS box) responsible for the interaction with the Gα subunit and GAP activity. The last one to be identified was RGS21, as a component of taste bud signaling [21,22]. In 2005, David P. Siderovski and Francis S. Willard summarized 37 human proteins containing RGS box classified into 10 subfamilies primarily based on sequence homology comparisons (Figure 2A) [19]. However, although some GPCR kinases (GRKs), sorting nexins (SNXs), etc., contain RGS homologous structural domains, most of them have not demonstrated GAP activity against Gα [23,24]. In the last decade or so, the structure, function, interaction with other molecules, expression, and regulation of RGS proteins have been richly studied and supplemented. According to the latest International Union of Pharmacology (IUPHAR) nomenclature, the RGS superfamily contains four subfamilies: RZ, R4, R7, and R12 (Figure 2B) [25].

The RZ subfamily members (RGS17, RGS19, and RGS20) act as negative regulators of multiple GPCR signaling, interact with Gα_i_ and Gα_q_ subunits, and are mainly associated with processes such as cell proliferation, mu-opioid receptor desensitization, and tumorigenesis (Table 1) [26,27,28]. The RZ subfamily members are highly homologous, with more than 50% amino acid identity. Compared to other RGS subfamilies, RZ subfamily proteins are small and relatively simple. Each member contains a short N-terminal polycysteine (Cys) string (Figure 2B). The Cys string acts as a palmitoylation substrate that anchors the protein to the membrane.

The R7 (RGS6, RGS7, RGS9, and RGS11) subfamily regulates neuronal differentiation in embryonic development [29,30] and is of interest for its key role in the regulation of a range of neuronal processes (e.g., mammalian vision, motor control, reward behavior, and injury perception) [31,32]. It is also involved in the pharmacological effects of analgesics for analgesia, tolerance, and addiction (Table 1) [33,34,35,36]. The four proteins of this group share three molecular modules: (i) the catalytic RGS structural domain, (ii) the GGL structural domain that recruits Gβ, (iii) the DEP structural domain that mediates the interaction with the membrane-anchored proteins R7BP and R9AP and the structural domain named DEP helix extension (DHEX) between the DEP and GGL structural domains (Figure 2B) [37]. The currently reported RGS R7-interacting Gα subunits include Gα_i_, Gα_q_, and Gα_12_ [38,39].

RGS10 is a small RGS protein that resembles the structure of the R4 subfamily members. However, based on the similarity of the RGS structural domain sequences, RGS10 is classified in the R12 subfamily. RGS10 is widely expressed in mammalian tissues and cells, with the highest expression in brain and immune cell subpopulations (Table 1). RGS10 selectively interacts with Gα_i_ proteins and is mainly associated with immune responses, platelet function, and inflammatory diseases of the immune system [40]. RGS12, the largest member of the RGS family, is abundant in the brain and osteoblasts, and is associated with neuronal differentiation [41], skeletal disorders [42,43], and cancer [44,45,46] (Table 1). RGS14 is highly enriched in hippocampal neurons, naturally inhibits synaptic plasticity, and limits learning and spatial memory [47,48,49], in addition to being associated with kidney disease (Table 1) [50,51].

The R4 subfamily is the largest RGS protein family, with 10 members. Each R4 family member contains only short N-terminal and C-terminal ends in addition to the RGS structural domain (except for RGS3) (Figure 2B). The N-terminal amphipathic helix present in most R4 family members binds phospholipids directly and plays an important role in membrane binding. The RGS structural domain is well conserved in R4 family members, whereas the N-terminal and C-terminal ends are different, which makes the non-GAP function specific. The R4 subfamily plays important roles in the hematopoietic system [52], the immune system [53], and the central nervous system [54]. Numerous recent studies have reported the role of R4 subfamily members in metabolic disorders, insulin secretion, and resistance (Table 1).

## 3. Regulation of Insulin Secretion and Uptake by Gα Proteins

β-cells are the predominant cell type within mammalian islets and are the sole source of circulating insulin. β-cells secrete insulin, which promotes the use and storage of glucose in the liver, fat, and skeletal muscle, ensuring blood glucose homeostasis. IR is a systemic disease in which cells do not respond to normal levels of circulating insulin. In this case, the metabolic functions of insulin, mainly those of the liver, muscle, and adipose tissue, such as glucose uptake and the synthesis of glycogen, lipids, and proteins, are disrupted.

Nutrient signals, such as glucose and amino acids, are the main triggers for insulin secretion by β-cells. When glucose enters β-cells, glucokinase initiates the glucose metabolism to increase the cytosolic ATP/ADP ratio [55]. An increase in the ATP/ADP ratio leads to the closure of KATP channels and membrane depolarization, which in turn opens voltage-gated calcium channels, resulting in an increase in intracellular Ca^2+^, which triggers insulin secretion [56]. Neuronal and hormonal signaling modulate insulin secretion by altering the activity and action of secondary messengers or effector molecules that control secretion [57]. GPCRs are major mediators of hormone and neuronal signaling, regulating insulin secretion.

Neurotransmitters or hormones bind to their respective GPCRs to activate G proteins, which then transmit regulatory signals by modifying the production of secondary messengers or interactions with effector molecules. All G protein subunits can transmit signals, with Gα being the main determinant of signal specificity and strength. All Gα family members are expressed in β-cells and are thought to be involved in the regulation of insulin secretion [58,59].

For example, cholecystokinin (CCK), glucagon, glucagon-like peptide-1(GLP-1), and PACAP activate Gα_s_, which subsequently stimulates cAMP production and enhances insulin secretion through protein kinase A (PKA)-dependent and independent pathways [60,61,62,63,64]. β-cell-specific Gα_s_ knockout mice exhibit reduced β-cell mass, increased β-cell apoptosis, and reduced islet size [65]. Activation of the Gα_q_ protein has also been found to enhance insulin secretion [66]. In contrast, Neuromedin U, galanin, somatostatin, ghrelin, and epinephrine activate Gα_i_ proteins and inhibit insulin secretion through calcium-dependent and calcium-independent processes [67,68]. These studies suggest an essential role of the Gα protein in regulating insulin secretion. In addition, the existence of different mechanisms also highlights the diversity and complexity of the roles and functions of Gα proteins in regulating insulin secretion. Kimple and Schaid et al. report that Gα_i/z_-null mice exhibit significant β-cell proliferation and increased β-cell mass, facilitating resistance to β-cell dysfunction caused by obesity [69,70].

In fact, each islet β-cell maintains thousands of insulin-secreting granules (SGs) at all times. Glucose stimulation induces the secretion of a small fraction of SGs, while promoting the biosynthesis of new SGs to maintain this reserve. The continued output of insulin can cause the process to fail. Studies have shown that the Gα_i/o_ protein can inhibit the release of insulin by inhibiting the docking of SGs with the β-cell membrane [71]. The inactivation of Gα_i/o_ results in the increased secretion of SGs [72].

In addition, Gα_q_ also plays a role in insulin signal transduction in adipocytes. Gα_q_ proteins translocate the insulin-responsive glucose transporter (GLUT4) by activating PI3K signaling downstream of insulin signaling [73]. The anti-Ga_q_ antibody can significantly reduce GLUT4 translocation in 3T3-L1 adipocytes [74], and the activation of skeletal muscle Gα_q_ signaling can improve skeletal muscle glucose uptake and systemic glucose homeostasis under physiological and pathophysiological conditions [75]. In skeletal muscle cell-specific Gα_q_-expressing mice, the receptor-mediated activation of Gα_q_ signaling promotes glucose uptake by the skeletal muscle and significantly improves glucose homeostasis in obese and glucose-intolerant mice. In contrast, severe deficits in glucose homeostasis occur in skeletal muscle cell-specific Gα_q_-deficient mice. In addition, the GPCR-mediated activation of Gα_q_ signaling also stimulates glucose uptake in primary human skeletal muscle cells.

**Figure 3:**
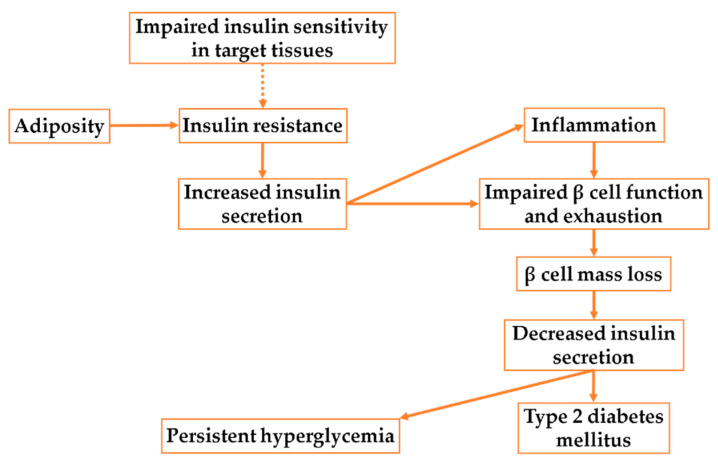
Schematic diagram of insulin resistance and the physiological and pathological consequences.

IR is the result of impaired insulin signal transduction by the insulin receptor itself, or any of its downstream effectors. Initially, peripheral IR causes pancreatic β-cells to secrete more insulin, known as compensatory hyperinsulinemia. Increased β-cell metabolic activity leads to the formation of reactive oxygen species (ROS) and endoplasmic reticulum (ER) stress, which induce inflammation. Low-grade local inflammation favors β-cell proliferation and insulin secretion. However, a sustained high level of insulin secretion leads to impaired β-cell function and exhaustion. Prolonged inflammatory mediators lead to the proliferation of resident macrophages and the recruitment of immune cells, further contributing to inflammation, impairing β-cell function, and causing exhaustion. Increased β-cell apoptosis gradually leads to the loss of β-cell mass, eventually leading to persistent hyperglycemia and T2DM [76,77] (Figure 3).

Studies have shown that the infusion of leucine can induce insulin resistance in experimental animals [78]. The exposure of cultured skeletal muscle cells, adipocytes, or hepatocytes to leucine induces insulin resistance [79,80,81]. In contrast, other studies have shown that leucine supplementation has no effect on the development of insulin resistance and may even improve insulin sensitivity and prevent diet-induced obesity [82,83]. These conflicting results remain unexplained. However, the experimental data of Yang et al. showed that leucine can promote insulin signal transduction in hepatocytes through the Gα_i_ protein [84]. Disregarding the paradoxical role of leucine, Leiss et al. clearly state that adipocyte-specific Ga_i2_-deficient mice also exhibit improved glucose tolerance and insulin sensitivity [85].

In summary, different isoforms of Gα proteins initiate different downstream signals and exhibit different cellular functions (Table 2). The activation of Gα_s_ promotes insulin secretion. In contrast, the activation of Gα_i_ inhibits insulin secretion, controls β-cell proliferation and mass, and suppresses insulin signaling in adipocytes. Gα_q_ signaling promotes insulin secretion and insulin signaling in adipose and skeletal muscle cells. It is essential to analyze the specific Gα protein of RGS protein action that would terminate different Gα signals.

## 4. The Potential Role of R4 RGSs in Insulin Secretion or IR

### 4.1. RGS1

Although RGS1 has been found to regulate the activity of the Gα_s_ homology protein MagA in *Magnaporthe grisea* [86], it is generally accepted that RGS proteins do not act directly on Gαs. Thus, RGS1 is thought of as a GAP for Gα_i2_ [87]. The two reported studies on the involvement of RGS1 in insulin resistance are superficial. In the study protocol of Choi et al., male C57BL/6 J mice (4 weeks old) were fed a low-fat diet (LFD) (D12450B, 16.17 kJ/g, 42% fat energy, 294% carbohydrate energy, and 84% protein energy) or a high-fat diet (HFD) (D12451, 19.866 kJ/g, 189% fat energy, 147% carbohydrate energy, and 84% protein energy) for 16 weeks [88]. Mice fed HFD exhibited obesity, insulin resistance, dyslipidemia, and adipocollagen accumulation. A microarray analysis of epididymis white adipose tissue (WAT) was performed in both groups, and a significant increase in RGS1 mRNA levels was found in the HFD mice (FC = 33.98, *p* = 5.75 × 10^−7^). In the study protocol of V. Moreno-Viedma et al., male low-density lipoprotein receptor knockout mice (*Ldlr*^−/−^, C57BL/6 J, 9 weeks old) were fed normal food or an HFD for up to 20 weeks [89]. Mice on the HFD exhibited obesity, insulin resistance, and atherosclerosis. A microarray analysis of the epididymis WAT of mice was performed, and a significant upregulation of RGS1 mRNA levels was found in the HFD group. The findings of these two studies are generally consistent, indicating an upregulation of RGS1 expression in the WAT of mice with obesity, insulin resistance, and dyslipidemia. However, the specific role played by the high expression of RGS1 remains unknown and is in need of further exploration.

### 4.2. RGS2

RGS2 typically binds to Gα_q/11_ to terminate its signaling [87,90,91]. RGS2 is the most widely studied protein in the R4 RGS family. The *RGS2* gene is located in a region on chromosome 1 (chr1.11:192,809,039–192,812,275) that is associated with body fat distribution and metabolic syndrome in men [92,93]. A prospective study randomly recruiting 2732 relatives and 348 unrelated individuals from 512 families from six European populations found that a C to G substitution at position S391 of the *RGS2* promoter increased RGS2 expression in adipocytes, correlating with European metabolic syndrome in white men [94]. Another cohort study recruited 406 (male, *n* = 294; female, *n* = 112) white hypertensive subjects (age 33 ± 9 years) and found that male patients with the *RGS2* 1114G allele had a higher body mass index (BMI) than those with the CC genotype [95]. However, this epidemiological relationship was not significant in the female patients.

*Rgs2*^−/−^ mouse models (C57BL/6 males) have been established, and primarily exhibit age-related weight control or obesity resistance. Dong et al. report that 8~10-week-old *Rgs2*^−/−^ mice exhibit similar body weight, serum glucose, and insulin levels compared to age-matched control wild mice [96]. Insulin tolerance assays also show similar insulin sensitivity. However, *Rgs2*^−/−^ mice exhibit more insulin secretion after glucose stimulation. In contrast, 25-week-old *Rgs2*^−/−^ mice exhibit a significant loss of pancreatic β-cells (70%) and significant reductions in serum insulin levels, weight, and epididymal fat compared to age-matched control wild mice. However, no differences are observed in fasting and non-fasting serum glucose or glucagon levels. The metabolic phenotype of older *Rgs2*^−/−^ mice is reported by Caroline et al. According to Caroline et al., 34-week-old mice continued to exhibit reductions in body weight and WAT, serum triglyceride, cholesterol levels, and serum insulin levels, but blood glucose levels were unchanged [97]. This phenotype will persist throughout the life of the mice until death (21–24 months). *Rgs2*^−/−^ mice exhibit greater levels of age-related weight control and obesity resistance, established from early to mid-life. The main reason for reduced insulin secretion after the early to middle period may be the significant loss of β-cells caused by *Rgs2* depletion. In vitro cellular experiments have also shown that *Rgs2* knockout β-cells are susceptible to hypoxia-induced apoptosis, whereas *Rgs2* overexpression protects β-cells from hypoxia-induced apoptosis [96]. Importantly, *Rgs2*^−/−^ mice consistently exhibit unaffected glucose homeostasis. The key reason for this glucose homeostasis remains to be further explored. However, targeting RGS2 may be beneficial in combating age-related obesity and even preventing T2DM.

RGS2 is considered to inhibit insulin secretion and insulin signaling and promote adipogenesis through its interaction with Gα proteins. RGS2 inhibits Gα_q_ signaling and blocks insulin-induced GLUT4 translocation in adipocytes [73]. The downregulation of RGS2 expression in adipocytes leads to increased insulin and catecholamine signaling, thereby enhancing glucose and fatty acid oxidation [98]. RGS2 has also been found to promote preadipocyte differentiation in a PPARγ-dependent manner [99,100]. However, the metabolic phenotype of *Rgs2*^−/−^ mice differs substantially from that of β-cell-specific Gα_q_/Gα_11_-deficient mice [101]. β-cell-specific Gα_q_/Gα_11_-deficient mice do not differ in pancreatic β-cell mass, number, or histology, but exhibit hyperglycemia and reduced early insulin secretion [101]. *Rgs2*^−/−^ mice are global knockout mice with multiple organ defects. Differences in systemic stress and β-cell-specific defects cannot be excluded. However, the metabolic differences between the three mouse models suggest the need to continue to explore the molecular mechanisms by which RGS2 acts, interacting with Gα proteins or with other molecules.

Direct evidence suggesting that RGS2 protein expression impedes insulin signaling in human cells and/or is associated with human T2DM comes from Vazquez-Jimenez et al. [102]. Vazquez-Jimenez et al. conclude that a 0.25 mM palmitic acid incubation is sufficient to induce insulin resistance in HUVEC-CS cells in vitro [103]. The upregulation of RGS2 protein expression is observed in HUVEC-CS cells that develop insulin resistance. Vazquez-Jimenez et al. suggest that RGS2 overexpression precedes insulin resistance, and that elevated RGS2 protein levels are sufficient to inhibit insulin signaling in human endothelial cells (or sufficient to trigger insulin resistance in human endothelial cells) [102]. Another study by Vazquez-Jimenez et al. reports a 3-fold increase in platelet RGS2 expression levels in T2DM patients compared to healthy people [102]. RGS2 expression levels in T2DM patients are significantly positively correlated with glycated hemoglobin (HbA1c) levels and negatively correlated with age and high-density lipoprotein cholesterol (HDL) levels. HbA1c level is an indicator of long-term glycemic control, and its percentage is also positively associated with a high risk of developing diabetes-related complications [104]. Unbiased principal component analysis shows that it is possible to distinguish healthy individuals from T2DM patients by HbA1c and RGS2 levels. Additionally, this negative correlation between RGS2 levels and age is explained by the fact that higher levels of RGS2 expression are found in younger T2DM patients. RGS2 expression again shows a correlation with age. Although this study includes a small number of subjects, including only 11 T2DM patients and 6 healthy individuals, the conclusions drawn need to be validated in a cohort with a larger sample.

In summary, mouse models and human cohort studies suggest the role of RGS2 in fat metabolism and deposition, and a correlation between age and gender is also exhibited. On the one hand, RGS2 could be indirectly involved in insulin resistance through the regulation of lipid metabolism. On the other hand, available data have also demonstrated that RGS2 can inhibit insulin secretion from β-cells, inhibit insulin signaling in metabolic cells, and maintain β-cell number and β-cell mass under stress, which are directly involved in T2DM. Future studies should perform experiments in animal models to validate the role of RGS2 in glucose tolerance and insulin sensitivity to demonstrate the value of targeting RGS2 in the treatment and prevention of T2DM.

### 4.3. RGS4

RGS4 interacts with Gα_i/o_ and Gα_q_ subfamilies [87,91,105]. RGS4 has extremely abundant transcript levels in mouse islets compared to other proteins of the same family [105,106,107,108]. Currently, several reports on the role of RGS4 in insulin secretion show contrasting results. Azua et al. constructed an RGS4 knockdown MIN6 cell line (mouse islet tumor cells) with *Rgs4*^−/−^ mice (systemic *Rgs4* knockout) and β-cell-specific *Rgs4* knockout mice, and in all of these in vitro and in vivo models found a significant increase in glucose-stimulated insulin secretion (GSIS) after *Rgs4* knockout [107]. Azua et al. suggest that RGS4 inhibits insulin release by suppressing M3 muscarinic receptors (M3Rs)/Gα_q_ signaling activation [107]. This conclusion is supported by the findings of Hu et al. [109]. In complete contrast, Iankova et al. reported that *Rgs4*^−/−^ mice exhibit increased serum catecholamine concentrations, increased circulating free fatty acid (FFA) concentrations, the abnormal accumulation of fatty acids in the liver and muscle, as well as elevated glucose levels, impaired glucose tolerance, and decreased insulin secretion [110]. However, they also showed that pancreatic β-cells isolated from *Rgs4*^−/−^ mice exhibit normal GSIS in vitro. Lankova et al. explained the defective insulin secretion in *Rgs4*^−/−^ mice as a toxic effect of FFA secondary to the hyperlipidemic phenotype. Lipid accumulation in the liver and muscle also inhibits insulin action in these cells, leading to insulin resistance. Interestingly, the data reported by Bastin et al. are different again, as they directly reported that *Rgs4*^−/−^ mice exhibit a significant deficiency in GSIS, both in mice and isolated islets [105]. Bastin et al. also report that *Rgs4*^−/−^ mice do not show significant differences in pancreatic β-cell mass, apoptosis, or SGs distribution compared to wild-type mice. Other studies have not reported results related to pancreatic mass. Bastin et al. interpret the differences as differences in genetic strategies and strains for *Rgs4*^−/−^ deletion, differences in mouse strain and size, and differences in GSIS experimental principles and methods. The role of RGS4 in insulin secretion needs to be validated and explained by further investigations.

### 4.4. The Other R4 RGSs

RGS3 is a GAP for the Gα_i_ and Gα_q_ subunit [111,112,113]. Raab et al. reported a transcriptome analysis of liver tissue from a mouse model (C57BL/6J, 3~5-week-old). The results show that a HFD (Harlan Teklad #TD88137, 42.16% fat, 42.81% carbohydrate, 15.02% protein, 4.53 kcal/g, and 10 weeks) suppresses Rgs3 expression in hepatocytes, but fasting for 48 h normalizes Rgs3 levels [114]. The results demonstrate a close dynamic correlation between RGS3 expression and nutritional intake.

RGS5 is a GAP for Gα_i_ and Gα_q_ [115]. Deng et al. reported that *Rgs5*^−/−^ mice (male, C57BL/6) exhibit hepatic fat accumulation, inflammation, impaired glucose tolerance, and significantly elevated circulating insulin levels [116]. Therefore, *Rgs5*^−/−^ mice exhibit insulin resistance and hyperinsulinemic compensation. Whether insulin resistance and hyperinsulin secretion compensation are the result of *Rgs5*-deficient-induced obesity or whether Rgs5 directly mediates insulin secretion and insulin signaling, as well as the effect of Rgs5 expression on β-cell mass and apoptosis, requires further experimental evidence.

RGS16 is a GAP for Gα_q_, Gα_i/o_ and Gα_13_ [20,117,118]. Vivot et al. reported an upregulation of *Rgs16* expression after glucose stimulation in rats, as well as isolated islets from rats [118]. The overexpression of *Rgs16* in mouse and human islets enhances GSIS and promotes β-cell proliferation, whereas *Rgs16* knockdown impairs GSIS and β-cell proliferation. This effect is thought to be mediated by somatostatin receptor/Gα_i/o_ protein/adenosyl cyclase/cAMP signaling [118].

## 5. Conclusions

**Figure 4:**
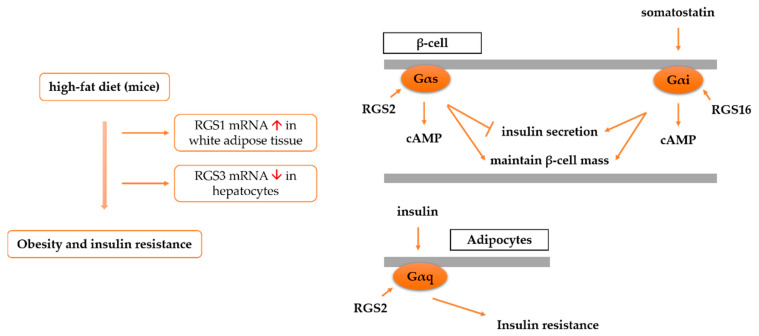
Signaling crossover of RGS R4 members in insulin secretion and insulin resistance.

R4 RGS members could play an integral role through interacting with Gα proteins in insulin secretion, insulin signal transduction, insulin resistance, lipogenesis and lipolysis, and β-cell function and quality. This review only provides a brief summary. The current study is relatively new and superficial (Table 3 and Figure 4). RGS2 is the most extensively studied member. On the one hand, RGS2 is associated with adipogenesis, obesity, and metabolic syndrome, and is also associated with insulin resistance by blocking insulin signal translation in adipocytes through interaction with the Gα_q_ protein. On the other hand, RGS2 can maintain islet β-cell function and quality by inhibiting insulin secretion and β-cell apoptosis. This may depend on the type of Gα protein that interacts with RGS2. Another extensively studied member is RGS4, with controversial results. There is also insufficient evidence for its results in promoting WAT lipolysis. RGS5 is found to be associated with abnormal liver fat accumulation, inflammation, and insulin sensitivity, and RGS16 is found to stimulate islet GSIS. Furthermore, the differential expressions of RGS1 and RGS3 are found in the tissue transcriptome analysis of HFD mice. Finally, the associations of RGS8, RGS13, RGS18, and RGS21 with insulin secretion or insulin resistance need to be further investigated. In conclusion, R4 RGS members play key roles in insulin secretion and insulin resistance and the related physiological processes. Continued in-depth research is necessary to facilitate the development of molecular drugs targeting R4 RGS to treat T2DM.

## Figures and Tables

**Table 1:** Brief functions of the RGS subfamily.

**Classification Basis**	Similarity of RGS structural domain sequences	
**Brief Functions**	RZ	Cell proliferation and tumorigenesis; mu-opioid receptor desensitization
	R4	Hematopoietic system; immune system; central nervous system; metabolic disorders; insulin secretion and resistance
	R7	Regulates neuronal differentiation in embryonic development; mammalian vision; motor control; reward behavior; injury perception; the analgesia, tolerance, and addiction of analgesics
	R12	RGS10: immune responses, platelet function, and inflammatory diseases of the immune system; RGS12: neuronal differentiation, skeletal disorders, and cancer; RGS14: inhibits synaptic plasticity and limits learning and spatial memory, kidney disease

**Table 2:** Summary of Gα subfamily members and their roles in T2DM.

Subfamily	Members	Roles	References
Gα_s_	Gα_s_ and Gα_olf_	Promote insulin secretion	[60,61,62,63,64]
Gα_i_	Gα_i1_, Gα_i2_, Gα_i3_, transducin-rod, transducin-cone, Gα_o-A_, Gα_o-B_, and Gα_z_	Inhibit insulin secretion; maintain β-cell number and function; insulin signaling in hepatocytes and adipocytes.	[67,68,69,70,71,72]
Gα_q_	Gα_q_, Gα_11_, Gα_14_, and Gα_16_	Promote insulin secretion; insulin signaling in adipocytes; skeletal muscle glucose uptake and systemic glucose homeostasis	[66,73,74,75]
Gα_12_	Gα_12_ and Gα_13_	N/A	

**Table 3:** Summary of R4 RGS subfamily members and their roles in T2DM.

Members	Roles	References
RGS1	Probably promotes insulin resistance	[88,89,114]
RGS2	Adipogenesis, adiposity, and metabolic syndrome in males; age-related obesity; blocks adipocyte insulin signal transduction via Gα_q_; inhibits insulin secretion by attenuating Gα_s_-cAMP signaling; maintaining pancreatic β-cell quality	[65,73,94,95,96,97,98,99,100,101,102,103,104,119]
RGS3	Unclear	[114]
RGS4	Insulin secretion	[105,107,109,110]
RGS5	Accumulation of hepatic fat; inflammation; insulin sensitivity	[116]
RGS16	Insulin secretion through somatostatin receptor/Gα_i/o_ protein/adenosyl cyclase/cAMP signaling	[118]
RGS8, 13, 18, and 21	N/A	
